# Cohort Trends in Working Life Expectancies at Age 50 in the United States: A Register-Based Study Using Social Security Administration Data

**DOI:** 10.1093/geronb/gbaa015

**Published:** 2020-01-31

**Authors:** Christian Dudel, Mikko Myrskylä

**Affiliations:** 1 Max Planck Institute for Demographic Research, Rostock, Germany; 2 Department of Social Policy, London School of Economics and Political Science, UK; 3 Department of Social Research, University of Helsinki, Finland

**Keywords:** Cohort study, Continuous working history sample, Foreign-born population, Working life expectancy

## Abstract

**Objectives:**

Little is known about the length of working life, even though it is a key indicator for policy-makers. In this paper, we study how the length of working life at age 50 has developed in the United States from a cohort perspective.

**Methods:**

We use a large longitudinal sample of U.S. Social Security register data that covers close to 1.7 million individuals of the cohorts born from 1920 to 1965. For all of these cohorts, we study the employment trajectories and working life expectancy (WLE) at age 50 by gender and nativity (native-born/foreign-born). For the cohorts with employment trajectories that are only incompletely observed, we borrow information from older cohorts to predict their WLE.

**Results:**

The length of working life has been increasing for the native-born males and females, and the younger cohorts worked longer than the older cohorts. However, WLE might soon peak, and then stall. The gap in WLE between the native-born and the foreign-born has increased over time, although latter group might be able to catch up in the coming years.

**Discussion:**

Our ﬁndings show that studying employment from a cohort perspective reveals crucial information about patterns of working life. The future development of the length of working life should be a major concern for policy-makers.

Increasing the length of working life is a major policy goal in many countries. In the United States, the Social Security retirement age has been increased from age 65 to age 66 for individuals born in 1943–1954, and it will increase further for the cohorts born in 1955 and in later years (Behagel & Blau, 2012). Yet how the length of working life has developed in general, and at older ages in particular, is not currently known. Speciﬁc transitions during working life have been studied extensively, such as the transition from retirement to work. In contrast, the question of how much accumulated time is spent in work at older ages has received less attention, despite its importance for policy-makers (Dudel & Myrskylä, 2017).

Like life expectancy, the duration of working life can be studied from a cohort perspective or from a period perspective (Leinonen, Martikainen, & Myrskylä, 2018). In the cohort perspective, the average length of the working trajectories of individuals born during a given time is considered, for example, the duration of the working lives of individuals born in 1940. In the period perspective, the conditions of a single year (or a few years) are assumed to prevail during the lifetime of a synthetic cohort, resulting in artiﬁcial working trajectories. While the period perspective is a useful summary measure for the conditions of a single year, it does not reflect the experiences of any real cohort (Loichinger and Weber, 2016).

For the United States, studies using the cohort perspective have been rare. The exceptions are studies conducted by Hayward and Grady (1990) and Hayward, Friedman, and Chen (1996) based on data from the National Longitudinal Survey of Older Men (NLS). These authors studied males aged 45 and older from the 1907–1921 cohorts, and covering the years 1966 to 1983. Since that period, the likelihood of working at older ages has changed considerably (Henkens et al., 2018). How the length of working life has developed for more recent cohorts is not known, though. Moreover, no results are available for females or for the foreign-born population, whose working trajectories differ from those of native-born males (e.g., Calvo, Madero-Cabib, & Staudinger, 2018).

In this paper, we use longitudinal U.S. administrative data from the Continuous Working History Sample (CWHS) to study for the ﬁrst time the length of working life at age 50 at the population level from a cohort perspective. The CWHS is a 1% sample of Social Security numbers (SSN) and the associated earnings trajectories. Using information on nearly 1.7 million individuals, we assess the employment trajectories at ages 50+ of the cohorts born from 1920 to 1965, and present the results by gender and nativity (native-born/foreign-born). We focus on two main measures of working trajectories: cohort age proﬁles of employment; and cohort working life expectancy (WLE), deﬁned as the expected total lifetime spent in employment from age 50 to age 74.

We focus on ages 50+, as employment at these ages has changed considerably over time (Gendell, 2008). Trends in employment at older ages are driven by many factors, including social policy reforms and increases in the retirement age; changing dynamics of exit from and (re-)entry into the labor force through, for example, phased retirement or unretirement; and economic conditions. Calculating cohort WLE at age 50 allows us to summarize the net effect of all these factors on working trajectories. Comparisons of cohort WLE across groups show to what extent differences in the factors driving working trajectories accumulate or cancel each other out.

This paper contributes to the literature on aging and employment in several ways. We provide the ﬁrst comprehensive study of U.S. cohort data for birth cohorts from 1920 onward using a large, high-quality sample of Social Security Administration data that has so far been largely untapped for aging research. Moreover, to the best of our knowledge, we are the ﬁrst to report (partly) extrapolated working trajectories for the United States, which offer a glimpse into the future. Finally, we provide results for the foreign-born population, an understudied but growing part of the U.S. population.

## Background

### Working at Older Ages in the United States

The labor force participation of older individuals since World War II can be broken down into two phases. In the first phase, labor force participation rates at older ages decreased for males, and were ﬂat for females. In the second phase, from the 1990s onward, participation rates increased for both older males and older females (Gendell, 2008). According to the Bureau of Labor Statistics (2017a), 46.2% of males aged 55 and older were part of the labor force in 2016, up from 38.3% in 1996. For females aged 55 and older, the corresponding figures were 34.7% (2016) and 23.9% (1996).

Several explanations have been offered for these trends, including changes in norms and preferences among cohorts (Fernández, 2013; Schirle, 2008); improvements in health, longevity, and educational attainment (Pleau & Shaumann, 2012); and social policy reforms. These reforms include major legislation in 1983 that increased the full retirement age from 65 to 66 and from 66 to 67, and measures that reduced beneﬁts for early retirement. In addition, lawmakers removed the earnings test beyond the retirement age (Gendell, 2008). Moreover, enhanced beneﬁts for those who delay claiming their beneﬁts beyond the full retirement age have been introduced. Nevertheless, Social Security reforms and changes in the composition of population explain only a modest share of the changes in labor force participation rates at older ages (Banerjee & Blau, 2016).

Economic conditions might also play a major role in shaping the working trajectories of older adults (Dudel & Myrskylä, 2017). While there have been several recessions since the 1980s, the eﬀects of these downturns on the labor market and on older adults were mostly moderate or short-lived (Cahill, Giandrea, & Quinn, 2015). However, the effects of the 2007–2009 recession might have been more severe. Farber (2011) found that unemployment increased sharply, and Coile and Levine (2011) reported that unemployed workers had a higher probability of retiring than employed workers during this recession. Men were more aﬀected than women by the crisis (Engemann & Wall, 2009). Whether the recession has had a signiﬁcant impact on the duration of working life among the aﬀected cohorts is unclear.

Research has shown that working after reaching the full retirement age has become more common in recent years, especially among men (Pleau, 2010). This observation is in line with the consistent ﬁnding that for many older people in the United States, the transition from work to retirement is not a one-time, permanent transition, but is instead made up of more complex sequences of part-time work or bridge jobs (Calvo et al., 2018), or returning to the labor market after retiring (Hayward, Hardy, & Liu, 1994). Retirement patterns diﬀer by gender, ethnicity, education, and other variables; with white, highly educated males most closely following the conventional pattern from full-time work to retirement (Flippen & Tienda, 2000).

An increasing share of the older population is foreign-born, and the proportion of immigrants in the workforce aged 55+ is rising. In 2017, this proportion was 15%, according to the Bureau of Labor Statistics (2017b). The working trajectories of foreign-born males differ from those of native-born males in several ways. First, the employment rates of native-born males tend to decline more sharply with age. Second, there is a cross-over age, before which the native-born have a higher likelihood of being in employment, and after which the foreign-born are more likely to be working. This pattern is partly attributable to the restrictions on eligibility for Social Security beneﬁts faced by immigrants (Borjas, 2011). Other potential reasons why immigrants may retire later than their native-born counterparts are they tend to have lower incomes (O’Neil & Tienda, 2015), and they are often in better health (Engelman, Kestenbaum, Zuelsdorff, Mehta, & Lauderdale, 2017).

### Working Life Expectancy

The length of working life has received far less attention in the literature than general and age-speciﬁc trends in labor force participation and employment. The small number of existing studies on the duration of working life used WLE as a main measure (Hoem, 1977; Loichinger & Weber, 2016). In this paper, WLE is deﬁned as the average lifetime spent in employment from age 50 to age 74, but in the literature, other age ranges are also used.

WLE has mostly been analyzed from the period perspective; that is, using artiﬁcial cohorts. Studies that have examined WLE from the cohort perspective are rare. The data demands for cohort studies are higher than those for period studies, as observing the working trajectories of a single cohort requires data covering many years. Moreover, as many cohorts have not yet reached the end of their working lives, their WLE is incomplete. The few existing studies on WLE include Leinonen and colleagues (2018) for Finland, Denton, Feaver, and Spencer (2010) using Canadian data, and Liefbroer and Henkens (1999) for the Netherlands. Leinonen and colleagues (2018) and Denton and colleagues (2010) also compared results from the period perspective with results from the cohort perspective, and found that they differed. It is unclear whether inequalities in WLE found using the period perspective are similar to those found using the cohort perspective.

The only studies for the United States that adopted a cohort perspective were conducted by Hayward and Grady (1990) and Hayward and colleagues (1996) using data from the National Longitudinal Survey of Older Men (NLS). Hayward and Grady (1990) studied men of the cohorts born from 1911 to 1921, and found that their WLE at age 55 was around 8.7 years. Hayward and colleagues (1996) reported, among other things, the partial WLE from age 55 to age 75. According to their estimates, WLE at these ages was 8.0 years for white men and 6.1 years for black men. This gap in WLE found between whites and blacks can be explained in part by diﬀerences in health and mortality.

In light of these ﬁndings on labor market trends and WLE, we can expect to find that the length of working life has been increasing among the more recent cohorts, and especially among females. This implies that the gap between males and females has likely narrowed. Moreover, we can expect to find that the recent reforms and the 2007/2008 recession are having clear effects on cohort WLE. It is, for example, likely that the recession slowed the increasing trend in cohort WLE. In addition, we can expect to observe that the increases in WLE are at least partly attributable to increasing employment at older ages. Making predictions for the foreign-born population is more diﬃcult. However, given the diﬀerences in the age patterns of employment between the foreign-born and native-born populations, we can expect to find that a larger part of the WLE of the foreign-born is at older ages, at least for males.

## Data and Methods

### Study Population and Measurement

Our longitudinal data comes from the Continuous Working History Sample (CWHS). The level of observation is the Social Security number (SSN). For each SSN, we have information on the earnings trajectory and any old-age pension and disability pension benefits that were received. The data comes from a 1% sample of all SSNs that covers the years 1970 to 2015 for the cohorts born between 1920 and 1965. The data includes information on individuals with SSNs from all U.S. states, as well as from Puerto Rico and other U.S. territories. For each SSN holder, we have information on their gender, birth year, and year of death, if applicable; and on whether the person was born in the United States (native-born) or in another country (foreign-born). We calculate the age as the year of observation minus the birth cohort. This means that the age variable is deﬁned as the age reached during a given year.

Throughout our analysis, we will assume that each SSN relates to a distinct individual. This is likely to be the case for most SSNs, but not all, as in rare instances one individual has several SSNs. For some of the individuals with several numbers, the CWHS includes an indicator that reports this information. As in most such cases, the individual’s earnings are associated with only one of his/her SSNs, while the other SSNs are associated with little or no earnings, we dropped all known multiple SSNs that are linked to little or no earnings. As the Social Security Administration issues multiple SSNs only in very speciﬁc circumstances, the number of these cases is small, for example, for the 1940 cohort, these individuals accounted for less than 0.5% of the sample. Thus, excluding these cases left the results virtually unchanged.

Employment is captured through annual earnings. We deﬁne an individual as employed for a given year if their earnings are above the threshold for a “quarter of coverage” for that year. A “quarter of coverage” (QC; or social security credit) is used to determine whether an individual is insured under the Social Security program. The threshold that must be reached to earn one QC has changed over time. Before 1978, for instance, a wage of 50 dollars or more for one quarter of the year was sufficient to earn one QC. But since 1978, when the reporting of earnings changed, QCs have been awarded based on annual earnings. The wage required to earn one QC was 250 dollars in 1978 and was 1,220 dollars in 2015. Alternative thresholds are discussed in the Supplementary Materials.

Before 1978, somewhat diﬀerent rules for earning QCs applied to the self-employed than to workers in dependent employment. To ensure consistency in our main analysis, we adopted the QC rules, and thus applied diﬀerent rules for self-employed and dependent earnings before 1978. Details and alternative analyses are presented in the Supplementary Materials. These analyses also include adjustments for the (rather minor) changes in the earnings coverage of the CWHS. The results of these analyses are very similar results to those presented here.

### Statistical Methods

For each cohort, we calculate the average number of person-years spent in employment by age. The person-years spent in employment are calculated by assuming that individuals with earnings above the threshold of one QC spent one full year in employment. Individuals who had earnings above the threshold, but who also either received retirement beneﬁts or disability beneﬁts or died during that year, are assumed to have spent a half year in employment. All of the other individuals are counted as having zero years in employment. Given the total number of person-years spent in employment age, the average number is calculated by dividing it by the cohort size at age 50.

WLE at age 50 is calculated as the sum of person-years spent in employment between ages 50 and 74, divided by the cohort size at age 50. We have chosen 74 as our upper limit because it is close to the limit used by Hayward and colleagues (1996), and it enables us to calculate the cohort WLE as for many cohorts as possible. Our results will be rather close to the actual cohort WLE because the number of individuals over age 74 who are in employment is extremely small, and contributes little to the overall WLE. More formal descriptions of the calculations are given in the Supplementary Materials.

To complete the WLE for the cohorts for whom the last observed age is below 74, we borrow information from the older cohorts, following Leinonen and colleagues (2018). If the time spent in employment at age x is not observed for one cohort, we take this information from the youngest cohort for which it is available. This method shows how the length of working life would develop if the conditions of the last period observed (2015) stayed constant. For the cohort born in 1942, only employment at age 74 was borrowed. Thus, the resulting WLE is likely very close to reality. For the 1965 cohort, on the other hand, only employment at age 50 is observed, and the resulting WLE strongly depends on the assumptions of this extrapolation approach. The WLE of the cohorts born from 1943 to 1964 is in between these two extremes. In addition to the approach by Leinonen and colleagues (2018), we implemented several alternative techniques to extrapolate employment at older ages. These led to qualitatively similar results (see Supplementary Materials).

Working trajectories and WLE are adjusted to take into account the possibility of an inﬂated cohort size due to unobserved outmigration. Outmigration is not captured in the data. We can also assume that deaths that occurred abroad are not captured, especially for the foreign-born population. For instance, if an individual migrates to the United States, works there for a certain period of time, and then returns to their home country, when the individual left the United States and when they died may not be recorded. Instead, the SSA record would simply show years with no contributions and with no beneﬁts received, potentially up to high ages.

To deal with this challenge, we exploit the fact that outmigration leads to the appearance in the data of “immortal” individuals who never seem to die. These cases can be detected by comparing the CWHS data with life table data from the Human Mortality Database (2018). Our comparison of these datasets showed that there are indeed too many surviving individuals in the data. We have removed these individuals from the sample. For details, see the Supplementary Materials. Alternative approaches also discussed in the Supplementary Materials led to similar findings.

## Results

### Sample Size

**Figure 1.**
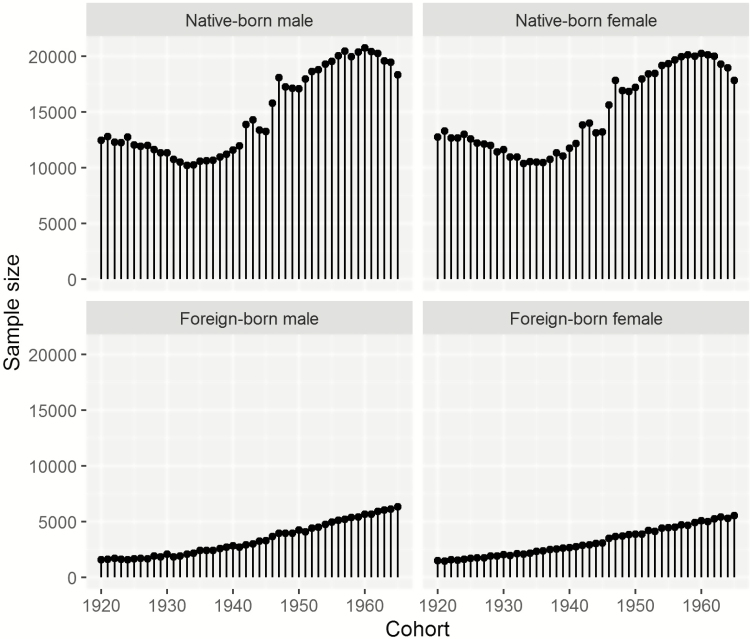
Sample size by cohort, gender, and nativity (native/foreign). Source: Continuous Working History Sample (CWHS); own calculations.

In Figure 1, the sample sizes for all birth cohorts by gender and place of birth are shown. In total, our analysis covers 1,675,011 individuals: 686,212 native-born males and 685,409 native born females; and 156,652 foreign-born males and 146,738 foreign-born females. Overall, 18% of the individuals in our sample are foreign-born. The sample sizes vary by cohort. For instance, for native-born men, the smallest sample size is 10,253 for the 1933 cohort, and the largest sample size is 20,783 for the 1960 cohort. The sample sizes are smaller for foreign-born individuals, with the smallest sample being 1,459 foreign-born females of the 1920 cohort. The sample sizes increase by cohort, and reach more than 5,000 for the foreign-born cohorts born in later years.

### Working Trajectories by Cohort and Age

**Figure 2.**
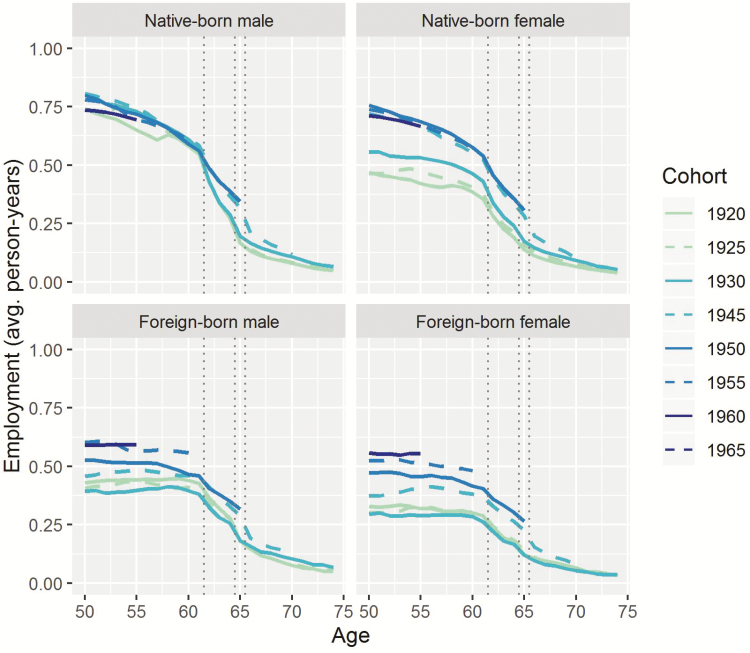
Cohort schedules of average person-years spent in employment, where each line represents a cohort. Older cohorts in light colors, younger cohorts in darker colors. The cohort trajectories for the younger cohorts are incomplete and miss older ages. Source: Continuous Working History Sample (CWHS); own calculations.

**Figure 3.**
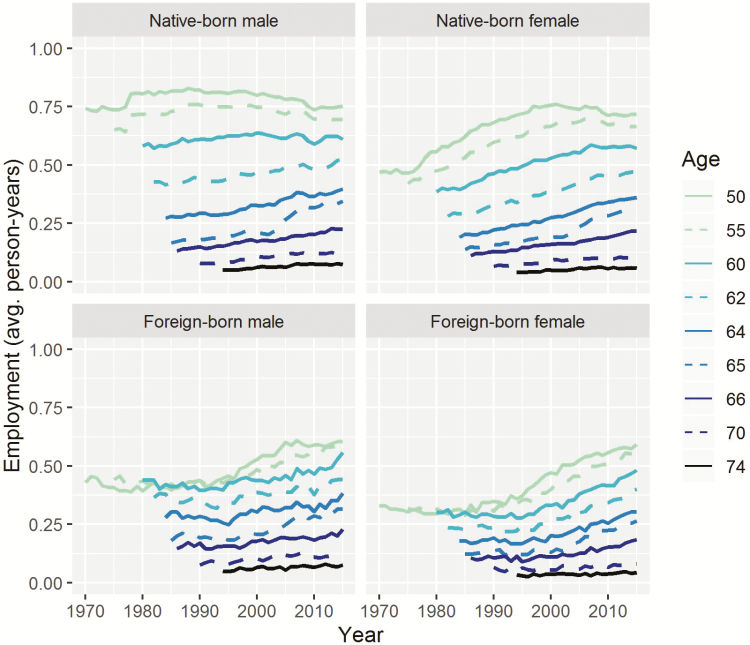
Trends in age-specific employment over time. Each line represents employment in a given age. Younger ages starting from age 50 are shown in light colors, older ages up to age 74 are shown in darker shades. Source: Continuous Working History Sample (CWHS); own calculations.

Figures 2 and 3 show results on employment trajectories by cohort and age. Speciﬁcally, Figure 2 shows age trajectories of average person-years spent in employment by gender and nativity (native/foreign). Each line represents selected cohorts from 1920 to 1965 (see the legend of the figure). The oldest cohort is shown in a light color (1920), and the youngest cohort is shown in a dark shade (1965). The cohorts born in 1920, 1925, and 1930 are observed up to age 74, while the younger cohorts are observed up to lower ages. The youngest cohort, born in 1965, is observed only up to age 50. Dotted vertical lines indicate important age thresholds: the youngest age pension benefits can be claimed (62), and the nominal retirement age (65 for cohorts up to 1937; 66 for cohorts 1943–1954). Figure 3 shows the same results arranged in a diﬀerent way, with each line representing one age (e.g., age 65), and showing how employment for that age evolved by calendar year. Ages starting from age 50 are shown in light colors, while older ages up to age 74 are shown in darker shades.

Looking at the figures, we can see that males generally had higher employment rates than females, and that native-born individuals generally spent more person-years in employment than the foreign-born. Employment varied strongly with age. It is also clear that employment after the retirement age—age 65 or 66 for the cohorts for whom employment around the retirement age is observed—increased for all groups, but was low in absolute terms and compared to employment levels at younger ages. Employment was very low after age 70.

For older native-born cohorts, employment during their 50s shows a discontinuity; for age-speciﬁc employment, this discontinuity is observed in the 1970s, with a jump occurring in 1978. This is due to the change in the rules for earning of quarters of coverage, as described in the data section. The eﬀect is less clear for the foreign-born. Results adjusted for this eﬀect are discussed in the Supplementary Materials.

For foreign-born individuals and native-born females, employment was higher overall in the younger cohorts, especially between age 50 and age 60. For females, increases in employment at these younger ages had stalled in recent years; while for the foreign-born, increases can be seen up to the last year observed (2015). For native-born males, by contrast, the cohort proﬁles seem to have tilted to the right over time, that is, the older cohorts had higher levels of employment than the younger cohorts between ages 50 and 55, while this pattern reversed starting at age 62 or 63. This means that the older cohorts worked more at younger ages, while the younger cohorts caught up at older ages.

These cohort proﬁles and age-speciﬁc employment levels clearly show the eﬀects of the nominal retirement age. For instance, for the cohort born in 1920, we see two breaks in the schedule: from age 61 to 62 (the lowest age at which benefits could be claimed), and from age 64 to 65 (the nominal retirement age). Between these breaks in the schedule, there was a rapid decline in employment. From age 65 onward, employment was low, and declined slowly but steadily. For the younger cohorts, we also see a break between ages 61 and 62, but the decline that followed was becoming less steep. Moreover, the break between ages 64 and 65 was shifting to age 66.

### Working Life Expectancy at Age 50

**Figure 4.**
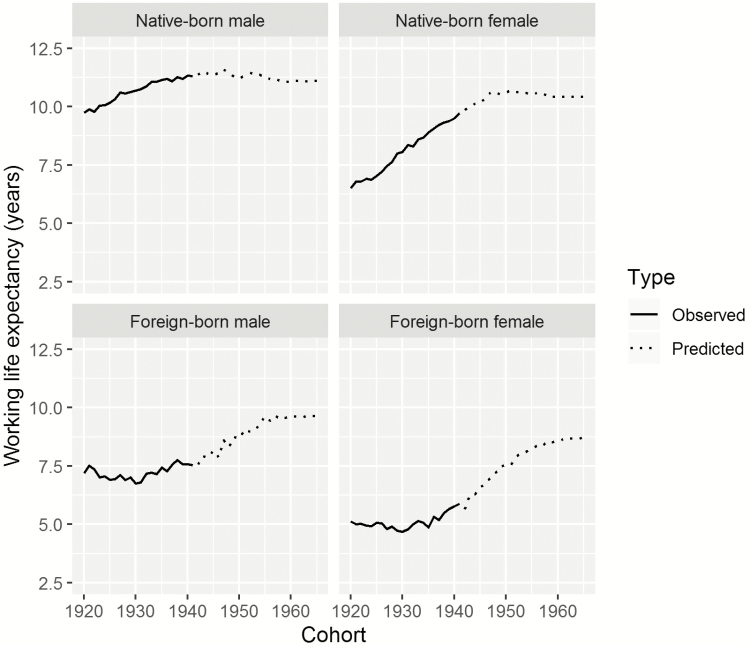
Working life expectancy (WLE) at age 50 by cohort, gender, and nativity (native/foreign). The solid lines are based on working trajectories ages 50 to 74. The dashed line represents results partly based on extrapolating recent cohort trajectories. Source: Continuous Working History Sample (CWHS); own calculations.

The results for WLE at age 50 by cohort are shown in Figure 4 and Table 1. Table 1 shows WLE and conﬁdence intervals for the total population by gender for selected cohorts, and by gender and nativity. The results for the total population closely follow those for the native-born, as the native-born make up at least 75% of the sample for all cohorts. Figure 4 displays the results for all cohorts. The conﬁdence intervals are not shown, as they are rather close to the point estimates, which is not surprising given the large sample size. The solid line in Figure 4 shows WLE at age 50 for the completely observed cohorts (1920–1941). The dashed line shows results that are based on the extrapolation approach described in the methods section.

**Table 1.** WLE in Years at Age 50% and 95% CI for Selected Cohorts by Gender and Nativity (native/foreign)

	Male			Female		
	CI			CI		
Cohort	WLE	95% lower	95% upper	WLE	95% lower	95% upper
Total Population						
1920	9.5	9.5	9.5	6.4	6.3	6.4
1925	9.8	9.8	9.9	6.8	6.8	6.8
1930	10.1	10.1	10.2	7.6	7.5	7.6
1935	10.5	10.5	10.6	8.2	8.2	8.2
1940	10.6	10.6	10.7	8.8	8.8	8.9
Native-born						
1920	9.8	9.7	9.8	6.5	6.5	6.5
1925	10.2	10.1	10.2	7.0	7.0	7.1
1930	10.7	10.6	10.7	8.1	8.0	8.1
1935	11.2	11.1	11.2	8.9	8.9	8.9
1940	11.3	11.3	11.4	9.5	9.5	9.5
Foreign-born						
1920	7.2	7.1	7.3	5.1	5.0	5.2
1925	6.9	6.8	7.0	5.1	5.0	5.2
1930	6.8	6.7	6.9	4.7	4.6	4.8
1935	7.4	7.3	7.5	4.9	4.8	4.9
1940	7.6	7.5	7.7	5.8	5.7	5.8

*Note*: CI = confidence interval; WLE = working life expectancy.

Source: CWHS; own calculations.

For the native-born male cohort of 1920, we ﬁnd a WLE of 9.8 years. Up to the 1941 cohort, WLE at age 50 increased by 1.5 years, to reach a total of 11.3 years. The forecasted results are only slightly above or below this number. They show some ups and downs, which reflect the diﬀerences in the partially observed working trajectories. The highest predicted WLE for native-born males is for the 1947 cohort, with a value of 11.6 years. For the cohorts born later than 1947, WLE declines. For the 1965 cohort, WLE is 11.1 years, which is roughly the same level as the WLE for the 1933 cohort.

The WLE at age 50 of native-born females increased considerably, and is predicted to level oﬀ after a small additional increase, as among recent cohorts, employment has stagnated at ages 60 and under. Speciﬁcally, the native-born females of the 1920 cohort had a WLE of 6.5 years, or 3.3 years lower than the WLE of their male counterparts. For the native-born females of the 1941 cohort, the WLE was 9.7 years. Thus, these women were catching up to the men, with the gap narrowing to 1.6 years. The WLE of native-born females is predicted to further increase to 10.7 years for the 1951 cohort, and then to slowly decline to 10.4 years for the 1965 cohort.

For all of the cohorts, foreign-born males and females had considerably lower WLE at age 50 than native-born males and females. Among the cohorts born from 1920 to 1941, WLE changed little for foreign-born males, and increased more than 5.5 years only for the foreign-born females of the 1938–1941 cohorts. This finding implies that the gap between the foreign-born and the native-born individuals increased slightly for males, and increased substantially for females. Speciﬁcally, the foreign-born females of the 1920 cohort had a WLE of 5.1 years, which was 1.4 years lower than the WLE of the native-born females. For the 1941 cohort, the WLE was 5.9 years for the foreign-born females and 9.7 years for the native-born females, which translates to a gap of 3.8 years between the native-born and the foreign-born.

The WLE of foreign-born males and females is predicted to increase. For instance, for the 1965 cohort, the WLE is predicted to amount to 9.6 years for foreign-born males and 8.7 years for foreign-born females, compared with 11.1 years and 10.4 years for their native-born counterparts, respectively. While the gap between native-born and foreign-born individuals appears to be narrowing, it is unlikely to close.

## Discussion

### Main Findings and Insights

Based on a large sample of administrative data, we present two major sets of substantive ﬁndings. The first set of results relates to working trajectories by age, and the second set is about the length of working life. Studying working trajectories and comparing older and more recent cohorts, we see that for native-born men, working trajectories have shifted toward employment at older ages, with employment at older ages (55+) increasing and employment at the younger part of our age range (50–55) decreasing; that for native-born women, employment has increased at all ages; and that for foreign-born males and females, employment has increased overall. With respect to the length of working life, we observe that working life expectancy (WLE) at age 50 has been increasing among the native-born, but might have reached a peak, and could level oﬀ in the future; while for the foreign-born, the duration of working life has remained mostly stable at a comparatively low level, but might increase if recent employment conditions prevail throughout the life course.

Two key insights emerge from our findings. First, differentials in age-specific employment by gender and nativity accumulate over the life course and lead to substantial differentials in the length of working life. Second, recent increases in employment at older ages do not necessarily translate to increasing WLE. Overall, our ﬁndings show that the future development of the length of working life should be a concern for policy-makers.

### Findings on Working Trajectories

The decrease in employment we found for native-born males below age 55 is in line with trends in labor force participation for that age group, and is attributable to the effects of the 2007/2008 economic crisis (Perez-Arce, Prados, & Kohli, 2018). The increase in employment at ages 55+ might partly reﬂect changes in retirement age, as the retirement age has increased to 66 for the cohorts born from 1943 to 1954. For native-born females, we found a constant increase in employment over the whole age range, in line with the general trend of increasing female labor force participation (Banerjee & Blau, 2016). Unlike for their male counterparts, there has been no decline in employment at ages below 55 for native-born females, possibly because women were less aﬀected by the 2007/2008 recession (Engemann & Wall, 2009).

One potential explanation for the shifting age patterns in employment of the foreign-born is that the composition of this population has changed with respect to the country of origin. Whereas in 1960, the majority of the immigrant population in the United States were of European origin, by 2010, the largest immigrant group came from Latin America (Grieco et al., 2012). Foreign-born Hispanics, who make up the largest share of immigrants from Latin America, have lower levels of educational attainment and English proﬁciency than their native-born counterparts (Read & Cohen, 2007), which might hinder their labor market outcomes.

A ﬁnding that is consistent across groups and irrespective of gender and nativity is that employment at ages older than the full retirement age has increased, but has remained low in absolute terms. After age 67, employment quickly drops to low levels, and is negligible after age 75. For instance, for the native-born men in our study, ages 65+ contributed 9% of WLE for the 1920 cohort, and 12% for the 1941 cohort, consistent with earlier ﬁndings on postretirement employment (Pleau & Shaumann, 2012). While postretirement employment is not uncommon, it is often part-time or for a limited period only (e.g., Calvo et al., 2018). As expected, we found that the contribution of ages 65+ to WLE was larger for the foreign-born than for the native-born: for the foreign-born males, employment beyond retirement age contributed between 13% (1920 cohort) and 18% of WLE (1941 cohort).

### Findings on the Length of Working Life

For the cohorts born from 1920 to 1941, for whom we fully observed WLE up to age 74, we found that WLE had changed very little, except among native-born females. Given the age-speciﬁc trajectories, these trends in WLE are not surprising, as age-speciﬁc employment increased considerably for native-born females. Additional analyses not reported here show that the eﬀects of changes in mortality on these trends are rather small. Compared to other countries, the United States has a high WLE at age 50. For instance, Leinonen and colleagues (2018) reported that the WLE at age 50 of the 1938 cohort in Finland was 7.0 years for males and 7.3 years for females. The corresponding values for native-born U.S. males and females born in 1938 were 10.6 years and 8.7 years.

Our forecasts of WLE for the cohorts born in 1942 and later indicate that for the native-born, a peak might have been reached or will be reached soon, despite policy eﬀorts to increase the length of working life, and despite increasing employment rates at older ages. For native-born men, this finding is mainly driven by the decrease in employment below age 55 discussed above. This suggests that a future increase in WLE—or even constant WLE levels—should not be taken for granted. For the foreign-born, on the other hand, the outlook is more optimistic, as the gap in WLE between this population and the native-born population might narrow. It is, however, unlikely to close.

### Methodological Considerations

The ﬁnding that WLE has been increasing or has remained constant for completely observed cohorts is in contrast to recent ﬁndings based on the period perspective, which showed no increase in WLE at age 50 and strong year-to-year ﬂuctuations (Dudel & Myrskylä, 2017). This is not surprising, as previous studies have shown that results from the cohort perspective differ from results from the period perspective (Denton et al., 2010; Leinonen et al., 2018), and we found similar discrepancies between the cohort and the period perspective in some of our additional extrapolation scenarios described in the Supplementary Materials. While results from the period perspective might be more timely, they tend to exaggerate the conditions that prevail over a period of 1 year or a few years. The ﬁndings for completely observed cohorts presented here describe patterns that people have actually experienced.

The extrapolation approach we use to complete working trajectories leads to rather robust findings. Specifically, for the cohorts born later than 1941, our estimates of WLE are partly based on the extrapolation approach by Leinonen and colleagues (2018), and the underlying assumption that incomplete working trajectories can be forecast by borrowing from the experiences of older cohorts. To assess the sensitivity of our results, we applied several alternative extrapolation methods. For instance, we forecasted WLE from the period perspective based on recent changes in employment. The alternative procedures all led to findings very similar to those reported in the results section, and to similar conclusions. Details are presented in the Supplementary Materials.

When interpreting our results, it is important to keep in mind that they are based on trajectories associated with Social Security numbers (SSNs). This creates three potential challenges. First, individuals who never applied for an SSN are not included in our data. Second, some individuals might have applied for and been issued several SSNs, across which the working trajectories of these individuals could be split. Third, outmigration is not captured in our data, which might be especially problematic when analyzing the foreign-born population.

The ﬁrst point—that is, that individuals who never applied for an SSN are not included in the data—is likely to be a negligible issue. Since only a small minority of the population does not apply for an SSN, their inclusion in the data would likely change the results only a little. Regarding the second point, individuals with multiple SSNs are ﬂagged in the data, and we adjust our analysis accordingly.

Third, outmigration is not captured. While living outside of the country, individuals might not have earnings in the United States, but could have earnings abroad. But since these earnings do not appear in the data, it may appear as if these individuals were not employed, when in fact they were. Dudel, López Gómez, Benavides, and Myrskylä (2018) found that the eﬀect of a similar issue in Spanish social security data was modest. To deal with this potential issue, we used data from the Human Mortality Database and remove excess survivors from the CWHS data (see Supplementary Materials for details). We also conducted several alternative adjustments and robustness checks. These analyses led to ﬁndings that are qualitatively similar to our main results, and to similar conclusions. Thus, it appears that our results are rather robust, especially with respect to differences between the native-born and the foreign-born, and with respect to trends over time. These analyses are also discussed in the Supplementary Materials.

## Supplementary Material

gbaa015_suppl_Supplementary_Material

## Notes

Author contributions: C. Dudel planned the study, performed all statistical analysis, and wrote the paper. M. Myrskylä helped to plan the study, including the statistical analysis, and to revise the manuscript.
