# Supplementary material for: Cohort Trends in Working Life Expectancies at Age 50 in the United States: A Register-Based Study Using Social Security Administration Data
Source: J Gerontol B Psychol Sci Soc Sci. 2020 Jan 31;75(7):1504–14. doi: 10.1093/geronb/gbaa015 (PMC7424272; doi:10.1093/geronb/gbaa015)
Supplement: gbaa015_suppl_Supplementary_Material [file gbaa015_suppl_supplementary_material.pdf]

Cohort Trends in Working Life Expectancies at Age 50 in the United States:

A Register-Based Study Using Social Security Administration Data

Supplementary materials

A Basic calculations and adjustment procedure

A.1 Notation

Let $n_c(x)$ denote the total size of cohort c at age x; e.g., $n_c(50)$ is the size of cohort c at age 50. $e_c(x)$ is the number of individuals of cohort c with earnings above the earnings threshold at age x. This covers three groups in the CWHS: individuals with earnings who do not receive Social Security benefits at that age and who survive until age $x+1$, $e_c^e(x)$; individuals who have earnings and also receive Social Security benefits and survive until age $x+1$, $e_c^s(x)$; and individuals who have earnings and die before reaching age $x+1$, $e_c^d(x)$, while potentially also receiving Social Security benefits.

A.2 Basic indicators

We use two indicators: the average person-years spent in employment by age, and the working life expectancy from age 50 to age 74. Without any adjustments, the average number of person-years spent in employment by age is given by

$$E_c(x) = \frac{e_c^e(x) + 0.5e_c^s(x) + 0.5e_c^d(x)}{n_c(50)}. \quad (1)$$

That is, individuals who receive only earnings contribute one person-year, while individuals who also receive Social Security benefits or die contribute one-half of a person-year. Working life expectancy from age 50 to age 74 of cohort c is given by

$$W_c = \sum_{x=50}^{74} E_c(x). \quad (2)$$

A.3 Adjustment for outmigration

As noted in the main text, outmigration is not captured by the data. This will affect $n_c(50)$ in two ways. First, some individuals might have outmigrated before reaching age 50, thus inflating $n_c(50)$. Second, individuals might outmigrate after age 50. Using $n_c(50)$ as the denominator in equation (1) is still valid in this case, and equation (2) will capture the remaining WLE in the U.S., while ignoring all employment abroad. It is, however, potentially problematic that the likelihood of outmigration differs for the native-born and the foreign-born, with the latter being more likely to leave the country, and the former being more likely to stay in the U.S. Thus, differences in WLE between these two groups will be due not only to differences in employment rates, but to differences in outmigration rates.

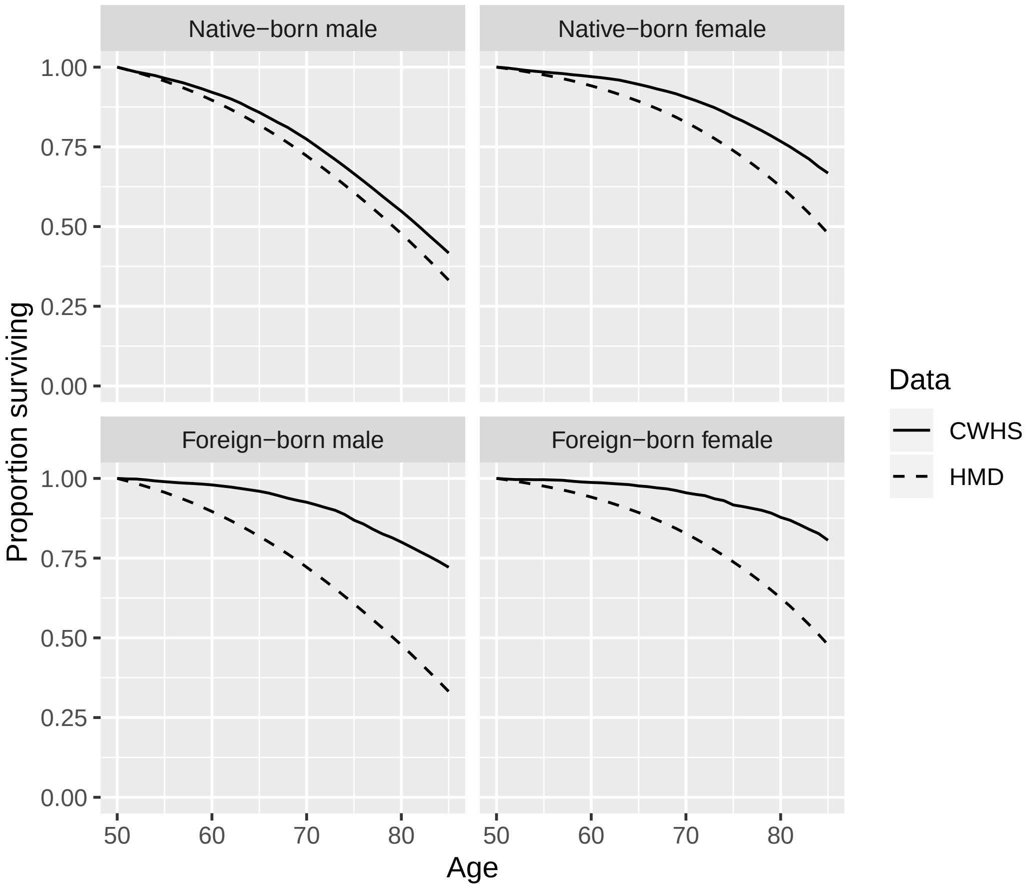

Figure 1: Survival of individuals born in 1930 from age 50 on in the CWHS (solid line) and in the HMD (dashed line, life table for the general population), by gender and place of birth. Survival in the CWHS is higher than in the HMD, due to outmigration. Source: CWHS; HMD; own calculations.

To deal with this issue, we exploit the fact that there are "immortal" individuals in the data who do not seem to die, as deaths abroad are often not registered in the data (see below for a discussion). Such cases can be detected by comparing the CWHS data with life table data from the Human Mortality Database (2018). Such a comparison is shown in Figure 1 for individuals born in 1930 by gender and nativity. The figure shows the proportion of surviving individuals by age starting from age 50 in both the CWHS data (solid line) and the HMD data (dashed line). The differences are striking for all of the groups. For the native-born men of the 1933 cohort, the HMD data indicate that about 33% survived to age 85, while the CWHS data show that 42% survived to this age. For the foreign-born men of the 1933 cohort, the differences are even more extreme, with the CWHS data indicating that 72% survived to age 85, and the HMD data showing that 33% survived to this age. The HMD estimates refer to the total male population, and thus provide only an approximate benchmark for the foreign-born

population. Nevertheless, the results clearly point to potential issues, and the differences between the groups suggest that there was considerably more outmigration among the foreign-born than among the native-born.

Formally, the adjustment works as follows. Let $s_c(x)$ be the survivor function for cohort c at age x taken from the HMD. Adjusted cohort size, $n_c^a(x)$, is then calculated as

$$n_c^a(x) = n_c s_c(x), \tag{3}$$

where n_c is the total cohort size for cohort c. Age-specific person-years in employment are then calculated as

$$E_c^a(x) = \frac{e_c^e(x) + 0.5e_c^s(x) + 0.5e_c^d(x)}{n_c^a(x)}. \tag{4}$$

WLE is calculated as

$$W_c = \sum_{x=50}^{74} E_c^a(x). \tag{5}$$

Essentially, individuals are removed once it becomes apparent that their survival is unrealistically high compared to the HMD data, but not earlier (see the next section for a discussion). Overall, this procedure leads to WLE estimates that are higher than the estimates without adjustment. The effect is rather small for most of the native-born cohorts, but the adjustment is greater for the foreign-born . Results without adjustment and using alternative adjustment procedures are discussed below, including results using alternative life tables for the foreign-born.

The adjustment procedure requires that deaths abroad are not registered in the data; i.e., it requires that there are differences between the data recorded in the CWHS and in the HMD. This is likely to be the case for a large fraction of the foreign-born who leave the U.S., as shown by the results in Figure 1; but it is less likely to apply to the native-born, especially if they are eligible for retirement benefits. The group of native-born individuals who permanently leave the country is likely to be relatively small, and should therefore not affect results very much. Temporary outmigration – that is, leaving the U.S. for shorter periods of time and then returning – is not covered by the adjustment procedure, but should not have a strong impact on WLE, as the number of individuals leaving the country for extended periods of time and then returning is also likely to be relatively small.

B Alternative adjustment procedures

The validity of the adjustment procedure described in the previous section depends on several assumptions. Here, we provide several alternative adjustment procedures that build on different and/or less strong assumptions. Comparing the results of the different procedures allows us to assess how sensitive our findings are with respect to the underlying assumptions.

B.1 No adjustment

As a reference for our main findings and for the alternative adjustment procedures described below, we calculated results without any adjustment based on the raw data. In this case, WLE is to be understood as expected WLE in the U.S., with potential additional WLE abroad that is not captured by the data.

B.2 Foreign-born with lower mortality than the native-born

It could be argued that the HMD life tables of the total U.S. population are not a good benchmark for the foreign-born, as the foreign-born tend to be healthier than the native-born (e.g., Engelman et al., 2017). Unfortunately, life tables by nativity are not available. As an alternative benchmark for the foreign-born, we used the life tables of Sweden as a scenario in which the survival of the foreign-born tends to be high. Otherwise, the adjustment procedure used was similar to the one described in the previous section.

B.3 Retroactive removal

Using the adjustment as in equation (3) removes individuals from the cohort once it becomes clear that the cohort size is too big when compared to reference life tables from the HMD. But outmigration leading to a surplus is a situation that could have happened before. In a sense, our adjustment procedure is not assessing the age-dependent timing of migration correctly, as it likely puts outmigration at ages that are too high. To deal with this issue, we calculated a scenario in which all surplus Social Security numbers (SSNs) are removed not from the age-specific cohort size, but from the total cohort size. This scenario is rather extreme, as it assumes that the individuals who are outmigrating leave as early as possible and do not contribute to employment. Together with the no adjustment scenario, this will lead to a range of results in which the true results will likely fall.

Technically, in this variant, we calculate the total number of surplus SSNs per cohort as

$$u_c = \sum_{x=50}^{\max_c(\text{age})} n_c(x) - n_c^a(x), \tag{6}$$

assuming that $n_c(x) \geq n_c^a(x)$. $\max_c(\text{age})$ is the maximum age up to which cohort c is observed. Cohort size as plugged into equations (4) and (5) is then calculated as

$$n_c^{a*}(x) = [n_c(x) - u_c]\, s_c(x). \tag{7}$$

B.4 Retroactive removal as per the 1920 cohort

Ideally, for the procedure described by equations (6) and (7) to identify the correct number of surplus $SSNs$, all of the cohorts should be observed up to an age at which all or most cohort members are dead. As this is not the case for some of the cohorts we study, the procedure will affect the results for different cohorts to differing degrees, depending on how high $\max_c(\text{age})$ is. For example, for the 1920 cohort, $\max_{1920}(\text{age}) = 95$, while for the 1941 cohort, $\max_{1920}(\text{age}) = 74$.

To test to what extent this might affect results, we applied the results for the cohort with the highest observed age (1920) to other cohorts by calculating the surplus SSNs as

$$u_c = n_c u_{1920}/n_{1920}, \tag{8}$$

under the constraint that $n_c^{a*}(x)$ as following from (8) and (7) cannot be higher than 50% of the number of inactive individuals (not employed, no receipt of Social Security benefits) of a cohort at age x. We took this approach for two reasons: first, individuals who were employed or receiving Social Security benefits in a given year might have left that year, but not earlier (ignoring multiple border crossings in and out); and, second, we can assume that at least some of the SSNs assigned to inactive individuals are real.

B.5 Adjustment for changes in SSP coverage and QC requirements

As we briefly discussed in the main text, the earnings covered by the CWHS changed over time, with the general tendency toward increasing coverage (Compson, 2011). For instance, before 1978, only earnings covered by the Social Security Program were covered; that is, the maximum taxable earnings for Social Security taxes. However, as the threshold of one quarter of coverage (QC) is considerably below the maximum

taxable earnings, this should not affect our analysis. There were also several other changes in earnings coverage, with coverage becoming more complete over time. Thus, the earnings data for more recent years should be complete or almost complete, and the employment estimates should be reliable.

In 1978, how QCs are awarded was changed. Before 1978, 400 dollars or more in annual earnings from self-employment earned four QCs. From 1978 onward, earnings from self-employment were treated as roughly the same as earnings from dependent work; i.e., in 1978, for 250 dollars of annual earnings, one QC was awarded, irrespective of whether the earnings were from self-employment or dependent employment. This change decreased the QC threshold for the self-employed. In our main analysis, we defined employment according to the rules applicable in a year, and thus used the 400-dollar threshold before 1978. This may have limited comparability across years.

To deal with these potential challenges in a consistent way, we used a simple procedure that works as follows. We calculated the ratios of the time spent in employment in consecutive years by cohort; e.g., the ratio of the time spent in employment in 1977 and 1978 for the cohort born in 1920. In a second step, we assumed that the ratio that occurs when coverage changed is indicative of the increase in coverage, and used it to scale up employment before the change.

The ratios as used in this approach can be seen in Figure 2 for the years from 1970 to 1981, shown by gender and by place of birth (native/foreign). Each solid line represents a cohort. Most values are below one, as in the age range we study employment generally decreases with increasing age, and thus from year to year. As can be seen for the native-born males and females, the ratio of the years 1977 and 1978 clearly stands out, and shows the effect of increased earnings coverage. The effect is roughly of the same magnitude for all depicted cohorts. We averaged this effect for all cohorts, arriving at a ratio of roughly 1.05 for the native-born males, and of 1.03 for the native-born females. This ratio was then used as a multiplier for the time spent in employment before 1978. For the foreign-born males and females, the effect is less clear.

This procedure was also applied to several other minor changes. The effect of the adjustment is, however, only notable for 1977/1978, which means that only the older cohorts are affected.

B.6 Four quarters of coverage instead of one

In our study, employment is defined as receiving enough income to earn a "quarter of coverage" (QC) under the Social Security program. This threshold is likely low, and should cover most employment. In 2015, for instance, $1,220$ dollars earned one QC.

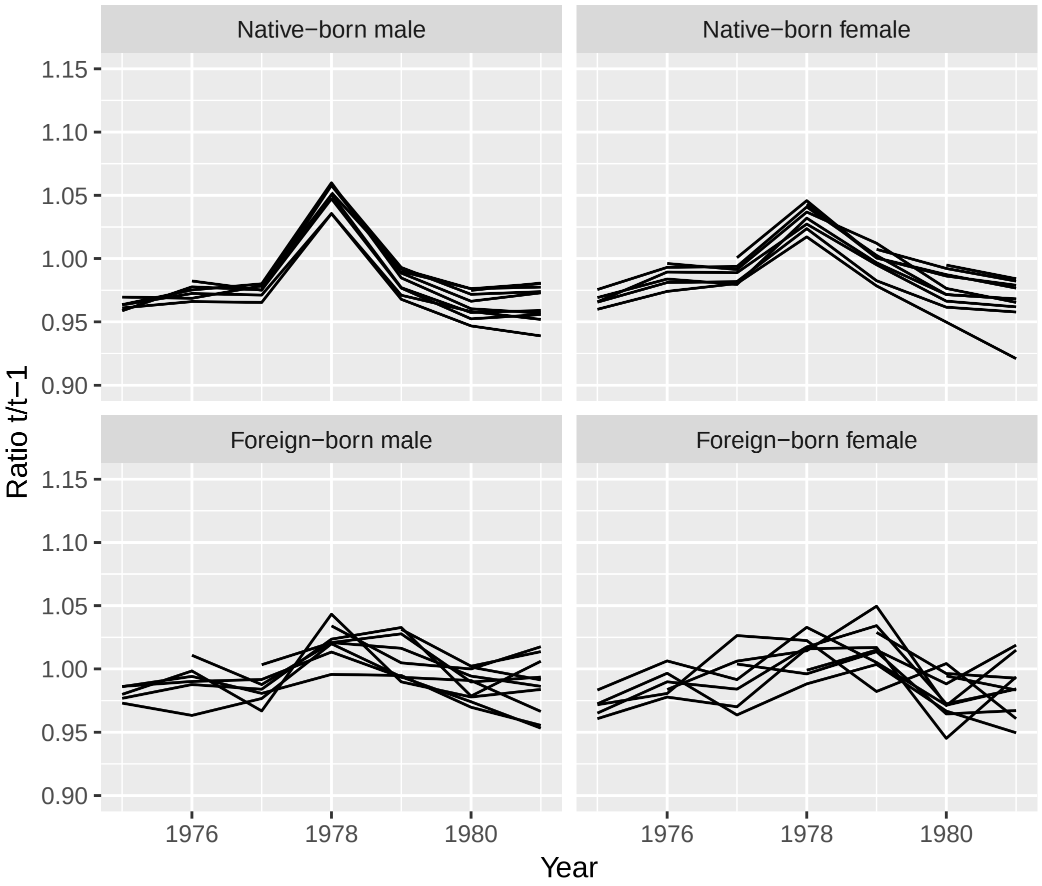

Figure 2: Annual differences in the time spent in employment by cohort for the years from 1970 to 1981, shown by gender and place of birth (native/foreign). Source: CWHS; own calculations.

This also means that part-time work and irregular work might count the same as full-time work, depending on how much income is earned. As an alternative, we used four QCs (1978: 1,000 dollars; 2015: 4,880 dollars) as a threshold instead of one QC. While this threshold is still low, it has two advantages. First, it should capture a large share of part-time work, which is very relevant for females and for older workers. Second, as the native-born tend to earn higher incomes than the foreign-born, high thresholds might capture the work of the native-born population, but not of the foreign-born population, which would bias the comparison between groups.

C Results of alternative adjustment procedures

The results of the additional analyses are captured in Figure 3. The upper-left panel shows the results by group from the main paper, and the other panels follow the order from this subsection; i.e., no adjustment (B.1), results using Swedish data (B.2), etc.

Overall, most of the results are close to our main findings. Especially the trends and the differences between groups seem to be extremely robust. The only partial exception to this pattern are the results based on the retroactive adjustment (section B.3), in which the trends differ somewhat. However, as this adjustment procedure affects the different cohorts to differing degrees (section B.4), this finding is not surprising, and is likely only an artifact of the adjustment procedure.

With respect to the level of WLE, the retroactive adjustment following the procedure described in section B.4 leads to levels that are considerably higher than our main findings. For instance, for the native-born males born in 1920, our main analysis estimates WLE at 9.8 years, while the adjusted result is about 11.8 years. Thus, the level of our main results could be biased downward. As the assumptions underlying the adjusted results are rather extreme, the differences between results show the maximum possible bias, which is unlikely to occur. The other adjustment procedures lead to results for which the levels are relatively close to our main findings.

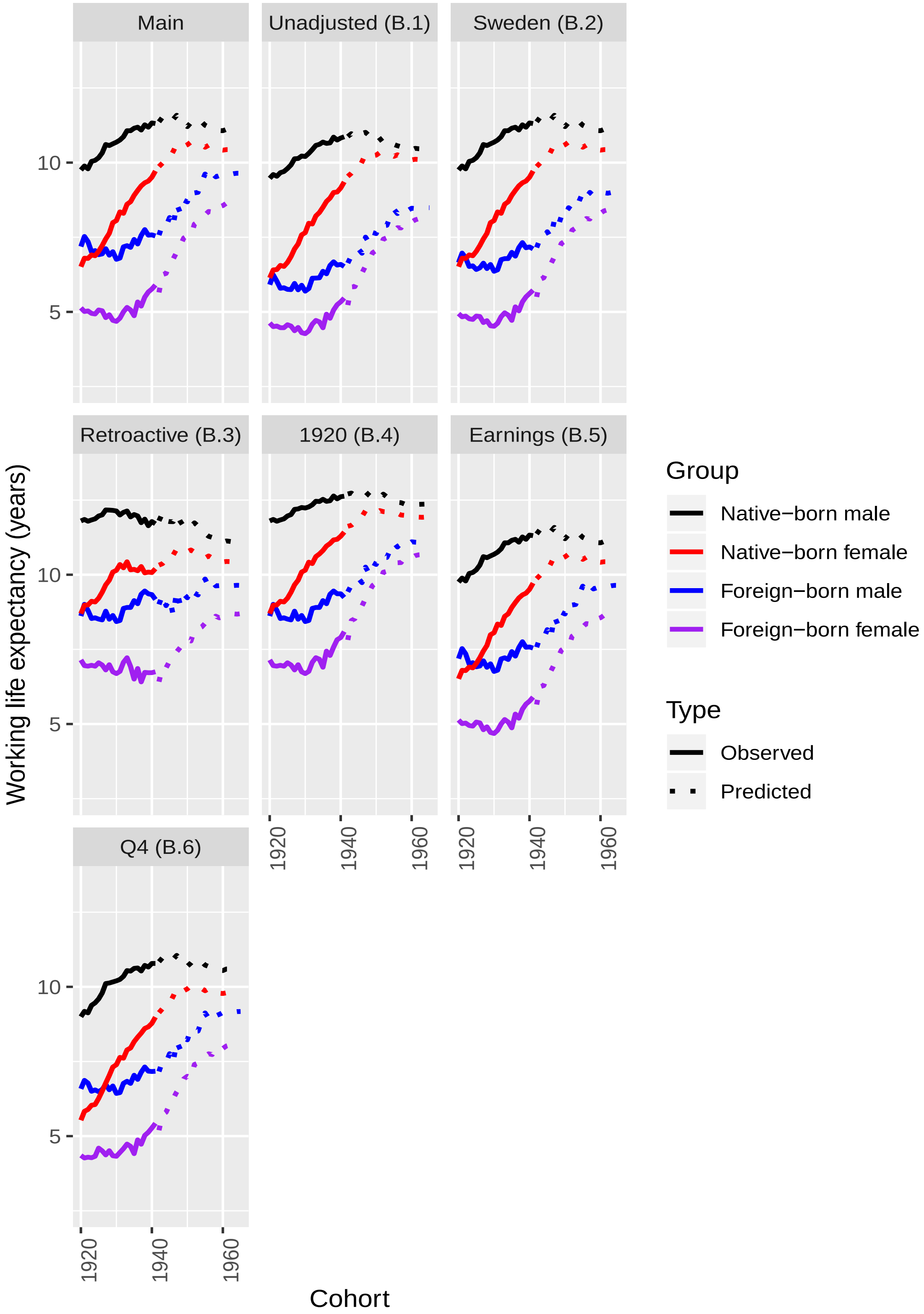

Figure 3: Results of the different adjustment variants. Source: CWHS; own calculations.

D Additional forecasts

Methods

For the cohorts for whom we do not observe the whole age range from 50 to 74, we use the extrapolation approach of Leinonen et al. (2018) to complete and forecast working trajectories. This approach rests on the assumption that the person-years spent in employment by age of the incompletely observed cohorts will be similar to those of the observed cohorts. Using this approach is likely unproblematic when it is applied to cohorts for whom only a small part of the working trajectory is missing, and relying on different assumptions about future employment rates will not affect the results much. On the other hand, for cohorts for whom the WLE estimates strongly rely on the forecast, alternative assumptions could lead to different results.

To assess to what extent our results depend on the assumptions that underlie the approach of Leinonen et al. (2018), we explored four alternative forecast approaches. The first two methods also use a cohort perspective for completing the working trajectories, while the third and fourth methods switch to a period perspective. All of these approaches lead to the same conclusion as the approach by Leinonen et al. (2018). Below, we also show examples of the working trajectories predicted by the different approaches, and we use one approach as an example to show how the same employment patterns can lead to different trends in the cohort perspective and the period perspective.

In the first, cohort-based approach, if for a given cohort and an age x the average number of person-years spent in employment is not observed, the absolute change in employment between ages x and $x-1$ is borrowed from the last observed cohorts. In the second approach, the relative change in person-years spent in employment between ages x and $x-1$ is used instead. For both approaches, we rely on data from the five last cohorts for whom ages x and $x-1$ are observed, and we take the average of those five values. We do not extend these approaches to older cohorts – e.g., the last 10 cohorts – as doing so would cover the impact of the financial crisis and extrapolate it to future cohorts, leading to declining WLE.

For instance, for the cohort born in 1942, the last observed age is 73 in 2015, and employment at age 74 is not observed. In the cohort-based approach using absolute differences, the absolute difference between person-years in employment at age 74 and at age 73 is calculated for each of the cohorts from 1941 to 1937, and is averaged. The resulting value is added to the person-years in employment at age 73 for the 1942 cohort to get the value for age 74. In the approach using relative differences, instead of the absolute change, the ratio of person-years in employment at age 74 and at age 73 is used,

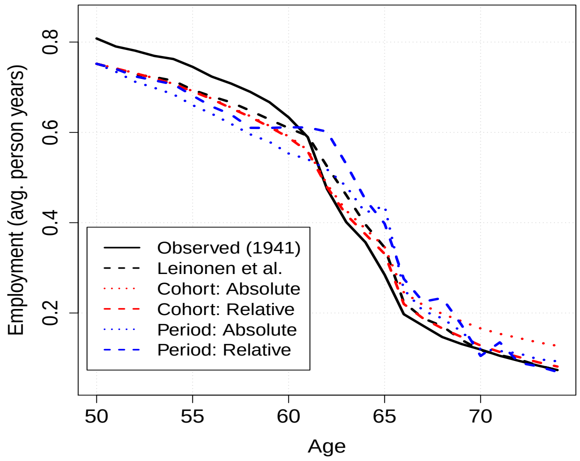

Figure 4: Forecast working trajectories for native-born men of the cohort 1965 by method; cohort 1941 as reference. Source: CWHS; own calculations.

and this ratio is multiplied with the person-years in employment at age 73 for the 1942 cohort to get the value for age 74.

The third and fourth approaches extrapolate employment from a period perspective. In the third approach, the absolute annual change in employment for age x is averaged over the last five years, and then repeatedly added to the last observed value for age x in 2015 to generate forecasts for age x in 2016, 2017, etc. The fourth method works in a similar way, except that it uses the relative annual change and repeatedly multiplies the last observed value. The youngest cohort for whom employment needs to be forecast is the 1965 cohort. This cohort reaches age 74 in 2039. Thus, in both the third and the fourth scenario, the forecast is conducted up to 2039, and the results are then rearranged to calculate WLE by cohort. Using the last 10 or 15 years instead of the last five years to calculate the absolute or the relative change applied in the forecasts does not lead to significantly different results.

Results: Working trajectories

To give an impression of to what extent the extrapolation approaches differ, Figure 4 shows the working trajectory for the 1941 cohort of native-born men as a reference (the last fully observed cohort), and the estimated working trajectory of the 1965 cohort resulting from the different extrapolation approaches. Age 50 is observed in the data for the 1965 cohort. With increasing age, the results of the different approaches slowly start to fan out, with the largest differences between the approaches being observed for ages 60+; although all of the methods predict increasing time spent in employment for

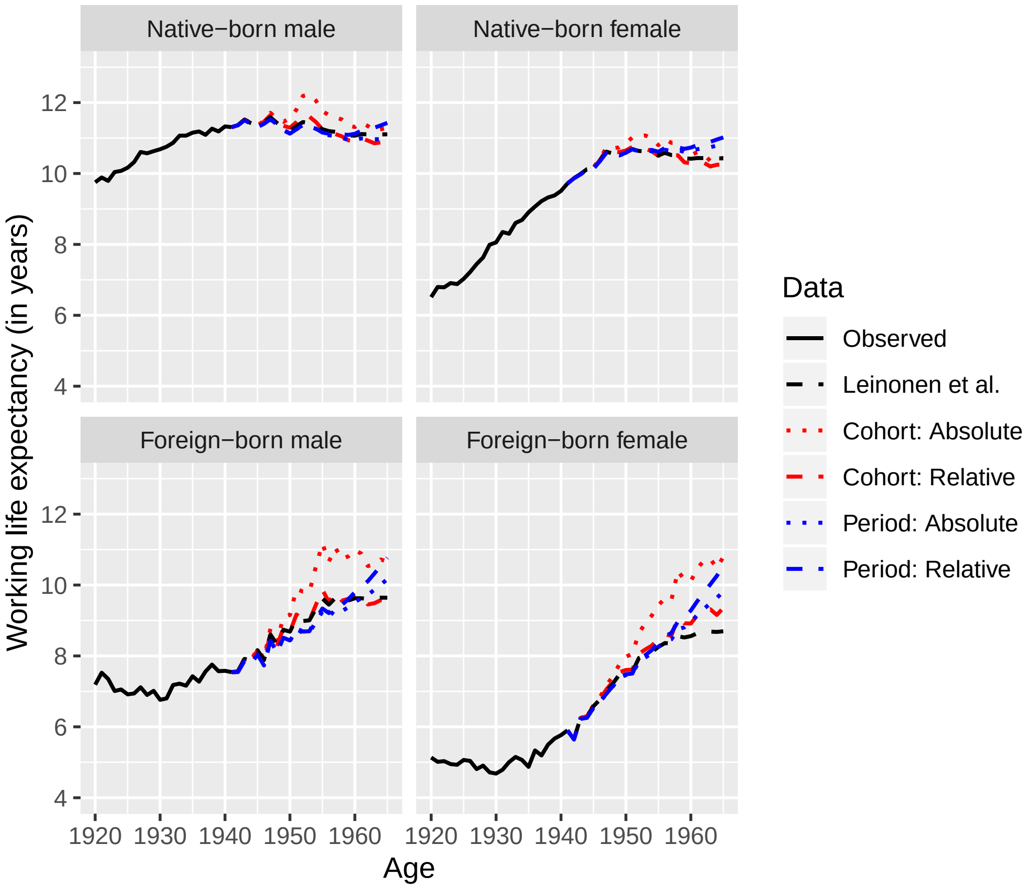

Figure 5: Working life expectancy at age 50 as resulting from the different forecast approaches. Source: CWHS; own calculations.

those ages. For instance, relative to the 1941 cohort, employment at age 65 is predicted to be between 16% and 54% higher for the 1965 cohort, depending on the method used. However, WLE at age 50 was 11.3 years for the 1941 cohort, and is predicted to be between 10.4 years and 11.4 years for the 1965 cohort, depending on the forecast method used. Thus, even though employment at older ages has been increasing considerably, all of the methods predict no, or only moderate, increases in WLE.

Results: Forecasts of WLE at age 50

Forecast trends of WLE at age 50 for all groups are shown in Figure 5. WLE for completely observed cohorts is shown as a solid black line. The forecast based on the approach of Leinonen et al. (2018), and that keeps employment fixed, is shown as a dashed black line. The results based on the cohort perspective are shown as red lines (dotted: absolute change; dashed: relative change), and the results based on the period perspective are shown in blue (dotted: absolute change; dashed: relative change).

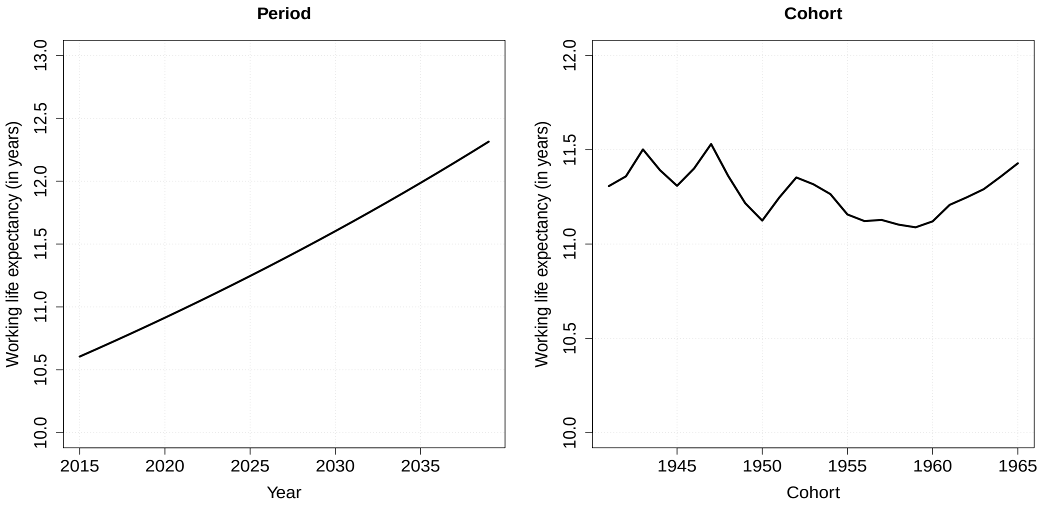

Figure 6: Results of period-based extrapolation, arranged in period perspective (left) and cohort perspective (right). Source: CWHS; own calculations.

For the native-born population, both male and female, all of the alternative forecast scenarios are consistent with our conclusions based on the method proposed by Leinonen et al. (2018): i.e., WLE is likely to peak soon and then stall, although when this peak will occur differs somewhat in different scenarios. A minor exception is the scenario that uses relative change from a period perspective, which shows a small increase in WLE after a period of stagnation.

For the foreign-born population, the differences between the scenarios are more pronounced, and both the pace and the levels of WLE increases differ. Still, all of the scenarios are consistent with the conclusions presented in the paper: i.e., the WLE of the foreign-born is likely to catch up to that of the native-born, at least to some extent.

Results: Cohort perspective vs period perspective

As we discussed in the main text, the results from the period perspective can differ from the results from the cohort perspective. Here, we show that the same employment trajectories arranged to calculate WLE at age 50 from the period perspective lead to results that differ from the results from the cohort perspective. Figure 6 shows this pattern for native-born men, based on the fourth extrapolation approach (period-based, relative annual change). The left panel shows the forecast from the period perspective, and the right panel shows the forecast from the cohort perspective. These results are based on the same employment trajectories for native-born males, just arranged and summed in different ways. In both cases, the first data point on the far left is (completely) observed. For the period-based forecast, this is the year 2015; and for the cohort forecast, this is

the 1941 birth cohort for whom all ages from 50 to 74 are observed. Years later than 2015 are forecast, up to 2039. For the cohorts born later than 1941, parts of the working trajectory are forecast. For the last cohort shown (1965), only age 50 is observed, and employment at all older ages is forecast.

The differences between the period and the cohort perspective arise because the results for each cohort cover experiences from many years, whereas the results for each year capture the experiences of many different cohorts. For instance, the results for the 1941 cohort cover the years 1991 to 2015, not all of which are covered in the panel on the right; while the results for the year 2015 cover the cohorts from 1941 to 1965.

References

Compson, M. (2011). The 2006 earnings public-use microdata file: An introduction. Social Security Bulletin, Vol. 71, No. 4.

Engelman, M., Kestenbaum, B. M., Zuelsdorff, M. L., Mehta, N. K., and Lauderdale, D. S. (2017). Work disability among native-born and foreign-born Americans: On origins, health, and social safety nets. *Demography*, 54(6), 2273–2300. https://doi.org/10.1007/s13524-017-0617-8

Human Mortality Database (2018). University of California, Berkeley (USA), and Max Planck Institute for Demographic Research (Germany). Available at www.mortality.org or www.humanmortality.de. Accessed 30 August 2018.

Leinonen, T., Martikainen, P., and Myrskylä, M. (2018). Working life and retirement expectancies at age 50 by social class: Period and cohort trends and projections for Finland. *Journal of Gerontology, Series B: Psychological Sciences and Social Sciences*, 73(2), 302–313. https://doi.org/10.1093/geronb/gbv104
